# Single-Bubble Rising in Shear-Thinning and Elastoviscoplastic Fluids Using a Geometric Volume of Fluid Algorithm

**DOI:** 10.3390/polym15163437

**Published:** 2023-08-17

**Authors:** Ahmad Fakhari, Célio Fernandes

**Affiliations:** 1 Department of Biophysics, University of Texas Southwestern Medical Center, 6001 Forest Park Rd, Dallas, TX 75390, USA; ahmad.fakhari@utsouthwestern.edu; 2 Transport Phenomena Research Center (CEFT), Faculty of Engineering at University of Porto (FEUP), Rua Dr. Roberto Frias s/n, 4200-465 Porto, Portugal; 3 Center of Mathematics (CMAT), University of Minho, Campus of Gualtar, 4710-057 Braga, Portugal

**Keywords:** geometric interface capturing approach, multiphase viscoelastic flows, OpenFOAM, elastoviscoplastic fluid

## Abstract

The motion of air bubbles within a liquid plays a crucial role in various aspects including heat transfer and material quality. In the context of non-Newtonian fluids, such as elastoviscoplastic fluids, the presence of air bubbles significantly influences the viscosity of the liquid. This study presents the development of an interface-capturing method for multiphase viscoelastic fluid flow simulations. The proposed algorithm utilizes a geometric volume of fluid (isoAdvector) approach and incorporates a reconstructed distance function (RDF) to determine interface curvature instead of relying on volume fraction gradients. Additionally, a piecewise linear interface construction (PLIC) scheme is employed in conjunction with the RDF-based interface reconstruction for improved accuracy and robustness. The validation of the multiphase viscoelastic PLIC-RDF isoAdvector (MVP-RIA) algorithm involved simulations of the buoyancy-driven rise of a bubble in fluids with varying degrees of rheological complexity. First, the newly developed algorithm was applied to investigate the buoyancy-driven rise of a bubble in a Newtonian fluid on an unbounded domain. The results show excellent agreement with experimental and theoretical findings, capturing the bubble shape and velocity accurately. Next, the algorithm was extended to simulate the buoyancy-driven rise of a bubble in a viscoelastic shear-thinning fluid described by the Giesekus constitutive model. As the influence of normal stress surpasses surface tension, the bubble shape undergoes a transition to a prolate or teardrop shape, often exhibiting a cusp at the bubble tail. This is in contrast to the spherical, ellipsoidal, or spherical-cap shapes observed in the first case study with a bubble in a Newtonian fluid. Lastly, the algorithm was employed to study the buoyancy-driven rise of a bubble in an unbounded elastoviscoplastic medium, modeled using the Saramito–Herschel–Bulkley constitutive equation. It was observed that in very small air bubbles within the elastoviscoplastic fluid, the dominance of elasticity and capillary forces restricts the degree of bubble deformation. As the bubble volume increases, lateral stretching becomes prominent, resulting in the emergence of two tails. Ultimately, a highly elongated bubble shape with sharper tails is observed. The results show that by applying the newly developed MVP-RIA algorithm, with a tangible coarser grid compared to the algebraic VOF method, an accurate solution is achieved. This will open doors to plenty of applications such as bubble columns in reactors, oil and gas mixtures, 3D printing, polymer processing, etc.

## 1. Introduction

The movement of individual and multiple gas bubbles within a liquid medium is a phenomenon that finds widespread relevance across a multitude of disciplines, including industrial processes, energy systems, environmental studies, food science, nuclear engineering, biological systems, and numerous other applications [1,2,3,4,5]. Extensive efforts encompassing experimental, numerical, and theoretical approaches have been undertaken to gain insights into the formation, shape, and dynamics of bubbles within a liquid medium [6,7,8]. Acquiring a deep understanding of this phenomenon plays a crucial role in the design and optimization of gas-liquid contact processes [9]. While previous studies have primarily focused on Newtonian fluids [10], the prediction of bubble rise becomes more intricate in complex fluids, where the relationship between strain and stress at the gas-liquid interface is non-linear. These materials which exhibit non-Newtonian fluid behavior are widely spread in cosmetics (such as shampoos, gels, and toothpaste), mud, suspensions, emulsions, slurries, foams, and polymers [10,11,12].

Viscoelastic fluid flow has garnered significant interest among researchers as a captivating subject within the realm of non-Newtonian fluids [13,14,15,16,17]. In the context of this flow regime, elasticity plays a crucial role in addition to the main parameters of gravity, gas-liquid surface tension, and liquid viscosity that dictate bubble rise in Newtonian fluids [18]. A key observation made by Astarita and Apuzzo [19] was that a bubble immersed in a viscoelastic fluid exhibited a sudden change in velocity when its size approached a critical volume threshold. The presence of this velocity jump discontinuity significantly amplifies the bubble velocity, leading to a transition in its shape from a convex configuration to a teardrop shape. Given the complex nature of viscoelastic fluids, it is highly advantageous to possess the ability to accurately calculate the progression of interfaces in such flows [20].

Multiple interface tracking methods have been employed for the analysis of viscoelastic multiphase flows. For example, the Front-Tracking Method (FTM), introduced by Unverdi and Tryggvason [21], is a Lagrangian approach that employs marker particles to track the interface within multiphase flows. The FTM provides explicit resolution of the interface, enabling precise representation of its dynamics and capturing detailed interface behavior with high accuracy. Sarkar and Schowalter [22] introduced an Alternating-Direction Implicit (ADI) front-tracking method, employing a finite difference scheme, specifically designed for simulating viscoelastic droplets in Newtonian fluids. Another paper Xia et al. [23] proposed a front-tracking/finite volume method for simulating the injection and subsequent cooling of hot polymer materials. In a subsequent study, Xia et al. [24] utilized the same numerical algorithm to conduct a three-dimensional simulation of fused filament fabrication. Another example of an interface tracking algorithm is the Marker-And-Cell (MAC) method [25], which is an Eulerian approach that employs markers to track the interface while being advected by the fluid flow. The MAC method utilizes a fixed grid to solve the Navier–Stokes equations, with the interface represented by a collection of markers that dynamically move in accordance with the fluid flow. Tomé et al. [13] introduced a numerical method for simulating viscoelastic free surface flow of an Oldroyd-B fluid. The governing equations were solved using a finite difference method on a staggered grid, inspired by the MAC method. In addition, a new formulation for computing the non-Newtonian extra-stress components on rigid boundaries was devised. The capabilities of the innovative technique were accessed in simulating various unsteady free surface flow problems. Recently, França et al. [14] focused on simulating the collision of shear-thinning and viscoelastic binary droplets, specifically addressing the dynamics of a two-dimensional free surface. To accomplish this, they employed a combination of two methods, FTM and MAC. This approach allowed for the accurate tracking of the droplets’ movement and the dynamic behavior of the free surface. By integrating the interface tracking capabilities of FTM with the grid-based calculations of MAC, França et al. [14] were able to provide valuable insights into the collision dynamics of shear-thinning and viscoelastic binary droplets, shedding light on the intricate interactions between the droplets and the surrounding fluid. In addition, Fernandes et al. [26] proposed an incompressible non-isothermal finite volume method to simulate the viscous flow of polymer melts. They specifically focused on the tracking of free surfaces in non-Newtonian inelastic fluids exhibiting shear-thinning and shear thickening behavior. The developed numerical method was utilized to accurately capture the flow characteristics of such fluids for the die-swell problem.

Despite the MAC method’s ability to accurately capture interface information [27,28], its computational cost is a significant drawback. This high cost arises from the need to consider a large number of virtual particles for interface tracking, which can be time-consuming. As a result, efforts have been made to develop alternative methods that strike a balance between accuracy and computational efficiency. Having that in mind, interface-capturing methods are frequently employed in the simulation of viscoelastic multiphase flows. One example of such a method is the Volume-Of-Fluid (VOF) [29], which defines the interface by identifying the volume occupied by each fluid with an indicator function. The VOF method relies on an advection equation to calculate the motion of the interface by solving for the volume fraction of each fluid. The VOF method has been extended to simulate non-Newtonian multiphase flows. For example, Fakhari et al. [30] measured the rheological parameters of three commercial inks for 3D printing, and these parameters were then fitted using Herschel-Bulkley and Sisko generalized Newtonian fluid models. Afterwards, two-phase fluid flow simulations of ink delivery in gravure printing using the VOF method were performed. Dynamic mesh refinement was also used to accurately capture the movement of the gravure cell. Different length scales and velocities were considered to assess the suitability of shear-thinning inks for several gravure cell sizes. Another example of an interface-capturing method is the Level-Set Method (LSM), a widely employed interface-capturing technique, that computes the interface by evolving a scalar function known as the level set function [31]. The level set function is defined such that it is positive inside one fluid and negative inside another fluid, and the interface is the zero level set of this function. Yu et al. [32] developed an LSM for incompressible, immiscible two-phase fluid flows, using a finite difference scheme on rectangular grids. The LSM was employed for simulating viscoelastic ink jetting, where the ink was modeled by the Oldroyd-B constitutive equation. In addition, Pillapakkam et al. [33] utilized the LSM to simulate the rise of bubbles in viscoelastic fluids, specifically focusing on determining the critical bubble volume at which the trailing end cusp emerges. Another paper from Li and Fangcao [34] studied numerically the polymer melt-filling process by using a coupled finite volume and LSM. The Immersed Boundary Method (IBM) has also been widely utilized for interface capturing in multiphase flow simulations [35]. The IBM represents the interface as an immersed boundary within a fixed grid [35]. This approach allows for the seamless integration of the interface into the computational domain, enabling efficient and accurate simulations of complex multiphase flow phenomena. By treating the boundary as an immersed entity, the IBM provides a flexible and robust framework for capturing interfaces in numerical simulations. The IBM utilizes a force-based approach to describe the interaction between the fluid and the immersed boundary. The boundary is typically represented as a set of discrete points or a continuous surface within the computational domain. The simplicity and flexibility of the IBM in generating meshes have made it a popular area of research, leading to the development of various advancements and novel features in different application domains [36]. Over the years, researchers have explored and implemented new techniques within the IBM framework to enhance its capabilities and expand its applicability in a wide range of scientific and engineering simulations. The IBM has also been used to simulate viscoelastic multiphase flows. One paper Saadat et al. [15] developed an immersed boundary algorithm using the finite element method. The algorithm, known as the immersed-finite-element method (IFEM), accurately determines the forces acting on solid particles. It was combined with the finite element method to simulate deformable Lagrangian solid particle suspensions on a fixed Eulerian grid. This combined approach can be applicable for simulating both Newtonian and viscoelastic fluids. Another paper Fernandes et al. [16] developed a fully resolved numerical solver for the simulation of solid spheres moving through viscoelastic fluids. The numerical algorithm was customized to enable the calculation of viscoelastic fluid flow and the corresponding hydrodynamic loads exerted on the particles. These loads determine the linear and rotational movements of the particles, which are then fed back to the fluid flow as moving no-slip boundary conditions applied to the particle surfaces. The Phase Field Method (PFM) is an alternative approach widely used for capturing interfaces in multiphase flows [37,38]. It employs a diffuse interface description, where the interface is represented by a scalar field that smoothly transitions between different phases [39]. The PFM has been extended to simulate viscoelastic multiphase flows, e.g., Li et al. [40] presented a PFM algorithm to simulate the deformation of biofilms, which are characterized with the Oldroyd-B constitutive equation. Zografos et al. [17] developed a two-phase viscoelastic solver based on PFM, and applied it to the simulation of an oscillating droplet. Recently, a unified PFM with two-way coupling for simulating fracture in viscoelastic materials was proposed by Dammaß et al. [41]. By considering the two-way coupling between the phase field and the mechanical response, the proposed unified PFM offers a comprehensive framework for accurately modeling fracture phenomena in viscoelastic materials.

Recently, Roenby et al. [42] introduced a novel geometric VOF method, known as isoAdvector, specifically designed for the advection of interfaces between two incompressible fluids. The isoAdvector method is implemented in the OpenFOAM computational fluid dynamics library [43]. However, it is known that at the interface reconstruction phase of isoAdvector, particularly for unstructured meshes, the adopted isosurface-based approach can introduce noisy interface orientations. Subsequently, Scheufler and Roenby [44] developed a computational interface reconstruction scheme based on the calculation of a reconstructed distance function (RDF), used to determine curvature instead of using the volume fraction gradient, coupled with a piecewise linear interface construction (PLIC) [45]. This way it is possible to achieve second-order convergence for both interface normal and position accuracy within cells [44]. This innovative scheme, PLIC-RDF, has been integrated with the interface advection step of the isoAdvector algorithm, further enhancing the accuracy and robustness of the method. This integration yields considerably reduced absolute advection errors, and it is possible to obtain second-order convergence for CFL numbers of 0.2 and below [44]. The PLIC-RDF isoAdvector method was subjected to various pure advection cases, demonstrating excellent performance in terms of volume conservation, interface sharpness, boundedness, and shape preservation [44]. Moreover, the implementation of the proposed interface reconstruction methods is straightforward and offers significantly decreased computational expenses compared to contemporary techniques, such as the algebraic VOF methods [46,47].

In this work, we extend the implementation of the multiphase PLIC-RDF isoAdvector algorithm to handle viscoelastic fluid flow calculations. The multiphase viscoelastic PLIC-RDF isoAdvector (MVP-RIA) algorithm was found to be a highly accurate method for capturing the intricate dynamics of interfaces between different phases. The method is based on the advection of a scalar function called the iso-surface, which is used to define the interface. The MVP-RIA algorithm uses an interpolation scheme to update the position of the iso-surface, which results in high accuracy and reduced numerical diffusion. In addition, the MVP-RIA algorithm is geometrically flexible and can handle complex topologies of the interface. Differing from the approach taken by Sun and Tao [48], Ling et al. [49], and Cao et al. [50], the PLIC-RDF isoAdvector algorithm uses a method of reconstructing the signed distance function within an interface cell that relies solely on information obtained from its adjacent points (i.e., cells it shares a vertex with). This approach enables the efficient implementation and parallelization of the proposed MVP-RIA algorithm, rendering it suitable for large-scale simulations. To the best of the authors’ knowledge, it is the first time that geometric PLIC-RDF isoAdvector algorithm is developed for multiphase viscoelastic and elastoviscoplastic fluid calculations, with more cost-effectiveness and accuracy compared to the algebraic VOF methods.

This paper is organized as follows. In Section 2, we provide the governing equations that describe multiphase viscoelastic flows of incompressible immiscible fluids. In Section 3, the numerical discretization used for solving the governing equations and the solution procedure of the MVP-RIA algorithm is detailed. In Section 4, we delve into three different case studies aimed at validating the MVP-RIA approach. First, we examine the buoyancy-driven rise of a bubble in a Newtonian fluid. Subsequently, the motion of a bubble in shear-thinning viscoelastic fluids was investigated. Lastly, we examine the buoyancy-driven rise of a bubble through an elastoviscoplastic material. The conclusions of the manuscript are shown in Section 5.

## 2. Governing Equations

In this study, we investigate an unsteady, laminar, isothermal, viscoelastic, and incompressible two-phase flow. The two fluid phases are assumed to be immiscible, i.e.,  without mass transfer across the interface. The governing equations for this flow are the mass conservation equation (Equation (1)),

(1) ∇·u=0,

and the balance of linear momentum equation (Equation (2)),

(2) $$\rho \frac{Du}{Dt}=-\nabla p+\nabla \cdot \tau +\rho g+f_{s},$$

where u is the velocity vector, ρ and *p* are density and pressure, respectively, $\frac{Du}{Dt}$ is the total time derivative of the velocity vector, g is the gravity acceleration vector, $f_{s}$ is the surface tension which will be explained in Section 3, and $\tau =\tau_{S}+\tau_{P}$ is the stress tensor given by the sum of Newtonian solvent contribution $\tau_{S}$ and polymeric contribution $\tau_{P}$. The solvent contribution reads as follows

(3) $$\begin{array}{c} \tau_{S}=\eta_{S}\left(\nabla u+\nabla u^{T}\right), \end{array}$$

where $\eta_{S}$ is the Newtonian solvent viscosity and the polymeric contribution $\tau_{P}$ is computed using a shear-thinning viscoelastic model given by the Giesekus constitutive equation [51] or by the elastoviscoplastic model given by the Saramito constitutive equation [52], defined, respectively, as 

(4) $$\begin{array}{c} \lambda {\overset{▿}{\tau }}_{P}+\tau_{P}+\frac{\alpha \lambda }{\eta_{P}}\tau_{P}\cdot \tau_{P}=\eta_{P}\left(\nabla u+\nabla u^{T}\right), \end{array}$$

(5) $$\begin{array}{c} e^{\frac{\varepsilon \lambda }{\eta_{P}}\mathrm{tr}\left(\tau_{P}\right)}\eta_{P}\max{\left(0,\frac{\bar{\sigma }-\tau_{0}}{k{\bar{\sigma }}^{n}}\right)}^{1/n}\tau_{P}+\lambda {\overset{\;□}{\tau }}_{P}=\eta_{P}\left(\nabla u+\nabla u^{T}\right), \end{array}$$

where λ is the fluid relaxation time, α is the mobility parameter responsible for shear-thinning behavior and quantifies the influence of the polymer chains’ stretch on the fluid’s viscosity, $\eta_{P}$ is the polymeric viscosity, $\eta_{0}=\eta_{S}+\eta_{P}$ is the zero-shear rate viscosity or total viscosity, ε is the extensibility parameter responsible for the elongational behavior and stretchability of the polymer chains in the viscoelastic fluid, tr(·) is the trace operator, max(·) is the maximum operator, $\bar{\sigma }=\sqrt{\frac{\tau_{D}:\tau_{D}}{2}}$ is the second invariant of the deviatoric stress tensor $\tau_{D}=\tau_{P}-\frac{\mathrm{tr}(\tau_{P})}{3}I$, the colon notation represents the double dot product, **I** is the identity tensor, $\tau_{0}$ is the yield stress, *k* is the consistency index, *n* is the flow behavior index and ${\overset{\;□}{\tau }}_{P}$ is the Gordon-Schowalter derivative given by

(6) $$\begin{array}{c} {\overset{\;□}{\tau }}_{P}\equiv {\overset{▿}{\tau }}_{P}+\zeta \left(\tau_{P}\cdot D+D\cdot \tau_{P}\right), \end{array}$$

where ζ is the non-affine deformation parameter, $D=1/2\left(\nabla u+\nabla u^{T}\right)$ is the rate of deformation tensor and ${\overset{▿}{\tau }}_{P}$ is the upper-convective time derivative of the polymeric extra-stress tensor defined as

(7) $$\begin{array}{c} {\overset{▿}{\tau }}_{P}\equiv \frac{\partial \tau_{P}}{\partial t}+u\cdot \nabla \tau_{P}-\tau_{P}\cdot \nabla u-\nabla u^{T}\cdot \tau_{P}. \end{array}$$

Note that a Newtonian fluid can be modelled using λ=0 and $\eta_{0}=\eta_{S}$ (no need to solve Equations (4) or (5) since $\tau_{P}=0$.)

In the continuum formulation, the simulation of high Weissenberg number flows in viscoelastic fluids is known to pose challenges in numerical convergence. These difficulties arise due to the exponential growth of stresses near critical points as the Weissenberg number increases. To address this issue, we adopt the log-conformation approach for computing the polymeric extra-stress tensor components in this study, following the implementation in the computational library OpenFOAM. The log-conformation approach, as described in previous works by Habla et al. [53] and Pimenta and Alves [54], offers a mathematical framework for effectively handling the complexities associated with viscoelastic flows. For a more comprehensive understanding of the log-conformation approach, the original works by Fattal and Kupferman [55] provide detailed explanations. Nevertheless, for the sake of clarity, the log-conformation approach starts with the definition of the relation between the positive definite conformation tensor (A) and the polymeric extra-stress tensor ($\tau_{P}$). Then, instead of solving the constitutive equation in A, it is reformulated in terms of the natural logarithm of A, θ=ln(A), leading to an evolution equation for θ. The Equations (4) and (5) written in terms of the natural logarithm tensor θ can be found in [56].

In our approach, we treat the two immiscible fluids as a single effective fluid across the entire domain. The physical properties of this effective fluid are calculated as weighted averages, taking into account the distribution of the volume fraction of each liquid. The equation governing the evolution of the volume fraction, denoted by γ, is given by

(8) $$\begin{array}{ccc} \frac{\partial \gamma }{\partial t}+\nabla \cdot \left(\gamma u\right) & = & 0. \end{array}$$

The original VOF method implemented in OpenFOAM (the so-called *interFOAM* solver) employed an artificial interface compression term $\nabla \cdot [\gamma \left(1-\gamma \right)u_{r}]$ [57] in Equation (8) to ensure the accuracy (sharpness) of the volume fraction field, and the MULES limiter (Multidimensional Universal Limiter with Explicit Solution) [58], to ensure boundedness of the volume fraction field. Here, $u_{r}$ is the vector of relative velocity between the two fluids. Although the *interFOAM* solver has been extensively employed in the past with successful results [30,57,59,60], it is known that, under certain conditions [61], the original VOF method in OpenFOAM may not be effective in preserving the desired sharpness of the interface. Moreover, the addition of the compressive velocity term is known to produce numerical artifacts during the interface advection.

On the other hand, the isoAdvector technique introduces innovative concepts in both the interface reconstruction and advection processes. The reconstruction step employs fast isosurface calculations to determine the fluid distribution within a grid cell. Meanwhile, the interface advection step utilizes a unique subdivision of the physical time step into smaller intervals, allowing for the analytical computation of volume fraction flux through a cell face. This is performed under the assumption that the interface is moving steadily across the face during the sub-interval. Details about the PLIC-RDF isoAdvector algorithm can be found in Roenby et al. [42], Scheufler and Roenby [44] and Gamet et al. [62].

## 3. Numerical Method

The VOF interface capturing method, initially proposed by Hirt and Nichols [29], utilizes a scalar function known as the volume fraction to track the interface between two immiscible fluids. In this representation, γ=1 corresponds to the region occupied by one of the fluids (e.g., Fluid A), while γ=0 corresponds to the presence of another fluid (e.g., Fluid B). Along the interface between the two fluids, the value of γ varies continuously within the range of 0<γ<1, indicating the transitional region. To ensure the accuracy of the numerical computations and to avoid the smearing of the volume fraction field while keeping it within the range of 0≤γ≤1, special attention is required. For that purpose, a technique for maintaining sharpness is utilized by adding an artificial interface compression term [57]. The MULES limiter is then employed to ensure that the volume fraction field remains bounded. For additional information on this methodology, refer to Deshpande et al. [58].

Furthermore, in the VOF interface capturing method, the presence of the interface between the two fluids is taken into account through the incorporation of surface tension in the balance of linear momentum equation (Equation (2)). This allows for the modeling of the interfacial forces that arise due to the surface tension effects, thereby capturing the behavior of the fluids at the interface more accurately. The surface tension at the interface generates an additional pressure gradient, resulting in a force, which is evaluated per unit volume using the continuum surface force (CSF) model [63]. The surface tension force, $f_{s}$, is calculated as follows [63,64],

(9) $$f_{s}=\sigma \left(\nabla \cdot \left(\frac{\nabla \gamma }{\left|\nabla \gamma \right|}\right)\right)\left(\nabla \gamma \right),$$

where σ is the surface tension coefficient, ∇γ=n is the normal vector to the interface [63], and the term in the middle of Equation (9) is the mean curvature of the free surface.

On the other hand, for the isoAdvector numerical algorithm, a piecewise linear interface construction (PLIC) [44] is employed along with a reconstructed distance function (RDF) [65] to calculate curvature, instead of using the volume fraction gradient. The iterative residual-based interface reconstruction procedure utilizing a reconstructed distance function to estimate the local interface position and orientation from the raw volume fraction data can be described in the following numerical steps:

1. Start by computing the raw volume fraction values for each cell in the computational domain using the VOF method.
2. Use the raw volume fraction data to generate an initial estimate of the interface position and orientation using a simple threshold operation. Cells with volume fraction values above a certain threshold (e.g., 0.5) are labeled as Fluid A, while cells with volume fraction values below the threshold are labeled as Fluid B.
3. Generate an initial estimate of the distance function from the interface [44]. This distance function is used to define an initial estimate of the interface normal and curvature at each cell.
4. Use the initial estimate of the distance function to calculate an RDF that better estimates the local interface position and orientation. Here, the gradient of the RDF is defined as the difference between the interface normal estimated from the distance function and the normal estimated from the initial threshold operation.
5. Update the interface position and orientation at each cell using the RDF, and repeat the previous step until the RDF converges to a desired tolerance.
6. Use the updated interface position and orientation to generate a new estimate of the distance function and repeat the previous steps until a desired level of accuracy is achieved.
7. Finally, use the updated interface position and orientation to calculate the interface curvature and normal at each cell, which can be used in subsequent calculations, such as interface advection or pressure-velocity coupling.

The numerical solution procedure of the multiphase viscoelastic PLIC-RDF isoAdvector (MVP-RIA) algorithm is represented in Algorithm 1. The MVP-RIA starts by initializing the fields (such as the velocity, pressure, viscoelastic stress tensor, and phase volume fraction (the latter one is performed using the *setFields* utility from *swak4Foam*)), setting the time step (or Courant number), the end time of the simulation and the number of PIMPLE [66,67,68] outer correctors (nOuterCorrectors) and pressure correctors (nCorrectors). The nOuterCorrectors enables iteration through the entire system of equations within a single time step. The algorithm enters a loop where it updates the face fluxes, updates the interface geometry using the PLIC-RDF isoAdvector method, computes the new viscoelastic stress tensor, computes the linear momentum equation and, lastly, enters a loop where nCorrectors sets the number of times it solves the pressure Equation (a Poisson type equation obtained from the mass conservation equation, Equation (1), and Rhie-Chow [69] interpolation) and momentum corrector. The PIMPLE outer correctors loop continues until the solution has converged or the maximum number of iterations has been reached. Finally, the algorithm outputs the results, including the velocity, pressure, viscoelastic stress tensor, and interface geometry fields.
**Algorithm1** Multiphase viscoelastic PLIC-RDF isoAdvector (MVP-RIA) algorithm**Require:** Mesh, physical properties, boundary conditions, initial conditions **Ensure:** Velocity, pressure, viscoelastic stress tensor and interface geometry fields 1: Initialize fields 2: Set time step, Δt, or Courant number, Co 3: Start time loop and set end time for simulation 4: Set the number of outer correctors, nOuterCorrectors, and pressure correctors, nCorrectors 5: Set current iteration count n=1 and pressure correctors count m=1 6: **while** not converged or n<nOuterCorrectors (PIMPLE corrector loop) **do** 7:    Compute face fluxes 8:    Update interface geometry using PLIC-RDF isoAdvector algorithm 9:    Compute viscoelastic stress tensor (Equations (4) or (5)) 10:    Compute linear momentum equation (Equation (2)) 11:    **while** m<nCorrectors (PISO corrector loop) **do** 12:      Solve the pressure equation and momentum corrector 13:      Increment iteration count *m* 14:    **end while** 15:    Increment iteration count *n* 16: **end while** 17: Output results

Second-order discretization schemes were employed for all calculations performed in OpenFOAM. Specifically, for the transient terms, the implicit Crank-Nicolson scheme was employed. For the gradient terms, the Gauss linear scheme was used, because only hexahedral meshes were constructed. In addition, the Laplace operators were discretized using the Gauss linear corrected scheme. Lastly, the advection terms in the linear momentum equation were discretized with the Gauss limitedlinearV1 scheme, which is specialized for vector fields and reduces to an upwind scheme in regions of strong velocity gradient, and the advection terms in the viscoelastic constitutive equations were discretized using the CUBISTA [70] scheme with component-wise and deferred correction implementation [54].

For the solution of the linear system of equations resultant from the discretized field equations, the following solvers were used: the generalized Geometric-Algebraic Multi-Grid (GAMG) linear solver [71] was employed for the discretized pressure equation (with tolerance equal to $10^{-10}$ and relative tolerance equal to 0.01), while the velocity and polymeric stress tensor linear systems are solved using BiCGstab with an Incomplete Lower-Upper (ILU) preconditioning (with tolerance equal to $10^{-8}$ and relative tolerance equal to 0) [72,73].

Following Gamet et al. [62], the simulations employed constant time steps with an average Courant number of 0.2 to keep the discretization errors resulting from the time scheme at a minimal level. For the PIMPLE algorithm configuration, we used nCorrectors=3, which corrected the pressure field three times in the PISO corrector loop, and nOuterCorrectors=5, ensuring five iterations of the pressure-momentum-stress coupling within a single time step.

## 4. Validation Case Studies

### 4.1. Buoyancy-Driven Rise of a Bubble in a Newtonian Fluid

**Figure 1:**
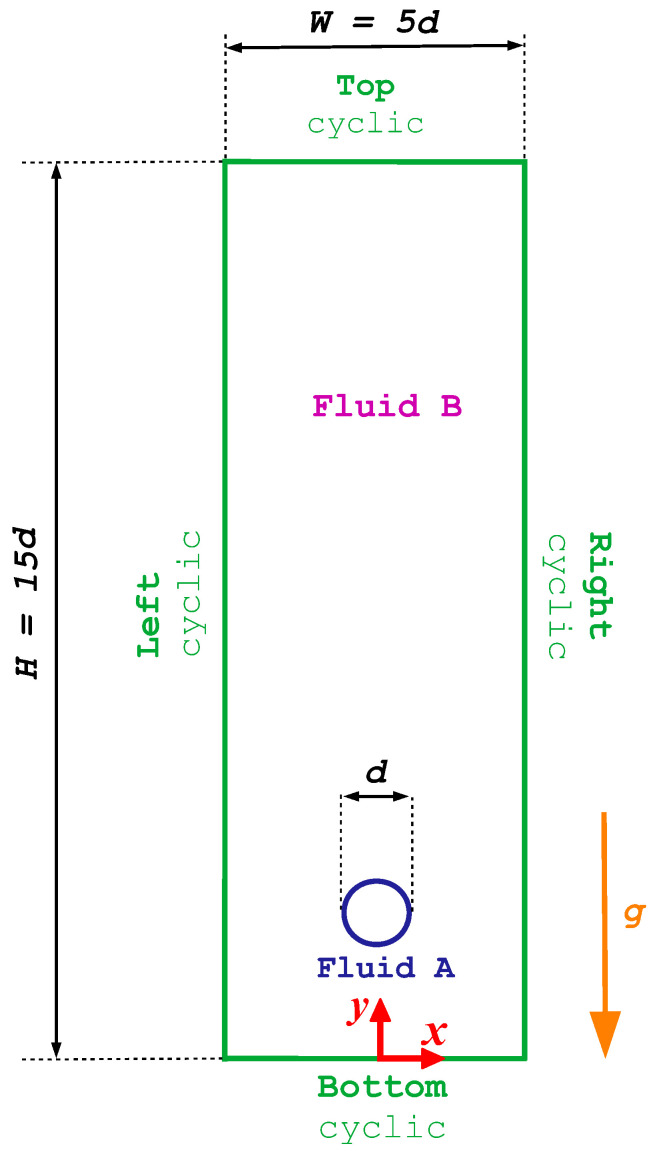
The geometry and boundary conditions for the buoyancy-driven rise of a bubble in a Newtonian fluid.

We examine the case study presented in Tsamopoulos et al. [7], which focuses on the buoyancy-driven rise of a bubble in a Newtonian fluid on an unbounded domain. The domain has a width of W=5 *d* in the *x* direction, where *d* represents the initial diameter of the bubble, and a height of H=15 *d* in the *y* direction (see Figure 1). A uniform square mesh with Nx=200 cells in *x* direction, and Ny=600 cells in *y* direction is generated, covering the initial bubble diameter with 40×40 cells. This domain and mesh are generated using the *blockMesh* utility. Figure 1 displays a sketch of the geometry and boundary conditions used for the simulation of the buoyancy-driven rise of a bubble in a Newtonian fluid. The boundary conditions at the top, bottom, left and right patches are of type *cyclic* for all the fields considered. The front and back boundaries of the domain are *empty* patches to be able to perform two-dimensional simulations. The bubble is initially located at a distance equal to 2 *d* from the domain’s bottom, left, and right boundaries.

For this case study, the dimensionless groups that arise from the governing equations are the Archimedes number, $Ar=\rho^{2}gR^{3}/\eta_{S}^{2}$, and the Bond number, $Bo=\rho gR^{2}/\sigma$, often called the Eötvös number, with R=d/2 being the initial bubble radius. From Ar and Bo dimensionless numbers it is possible to define the Morton (Mo) number, which relies solely on the physical properties of each liquid,

(10) $$Mo=\frac{Bo^{3}}{Ar^{2}}=\frac{g\eta_{S}^{4}}{\rho \sigma^{3}}.$$

Table 1 presents the fluid properties of liquids B-1 and B-2, which are used to describe the surrounding fluid (Fluid B) of the bubble. The corresponding values of Mo for B-1 and B-2 are $2.174\times 10^{-7}$ and $3.769\times 10^{-4}$, respectively. Fluid A, which represents the bubble, is defined by the dynamic viscosity and density properties of air.

**Figure 2:**
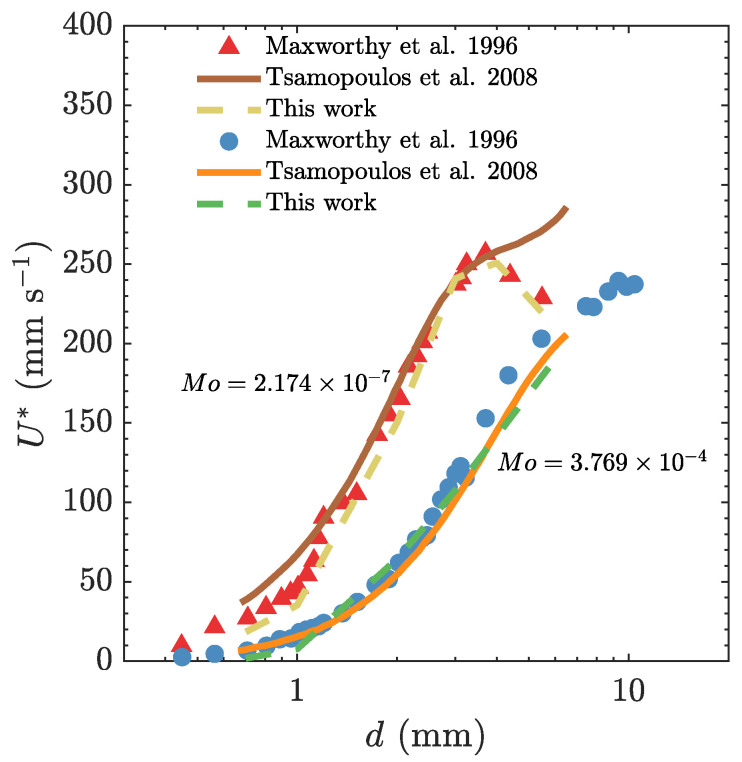
Comparison of our predicted bubble rise velocity with experimental data reported by Maxworthy et al. [6] and numerical simulation results by Tsamopoulos et al. [7]. The comparison is performed for two selected values of Mo number, representing different flow conditions in a Newtonian liquid.

Figure 2 illustrates the comparison of our calculated rise velocity $U^{*}$ as a function of bubble diameter *d* with experimental and numerical data available in the scientific literature [6,7]. Each data set corresponds to two different values of Mo number. In general, the simulations’ result for the lower Mo number follows the same trend shown by Maxworthy et al. [6]. The bubble velocity enhances as the bubble enlarges, while for the bubble diameters of d>4 mm, the bubble slows down with growth in size. In this case of the lowest Mo number, discrepancies from the Tsamopoulos et al. [7] results are found at larger bubbles. The reason for this difference can be explained by the fact that the maximum in the bubble velocity vs. bubble diameter corresponds to the minimum in a drag coefficient vs. Reynolds number curve that has been reported for these and other low-Mo fluids in the literature [6]. The simulations related to the larger Mo number, which corresponds to the more viscous fluid, predicted the bubble velocity in very good agreement with both experiments by Maxworthy et al. [6] and simulations by Tsamopoulos et al. [7] for all the bubble diameters.

**Figure 3:**
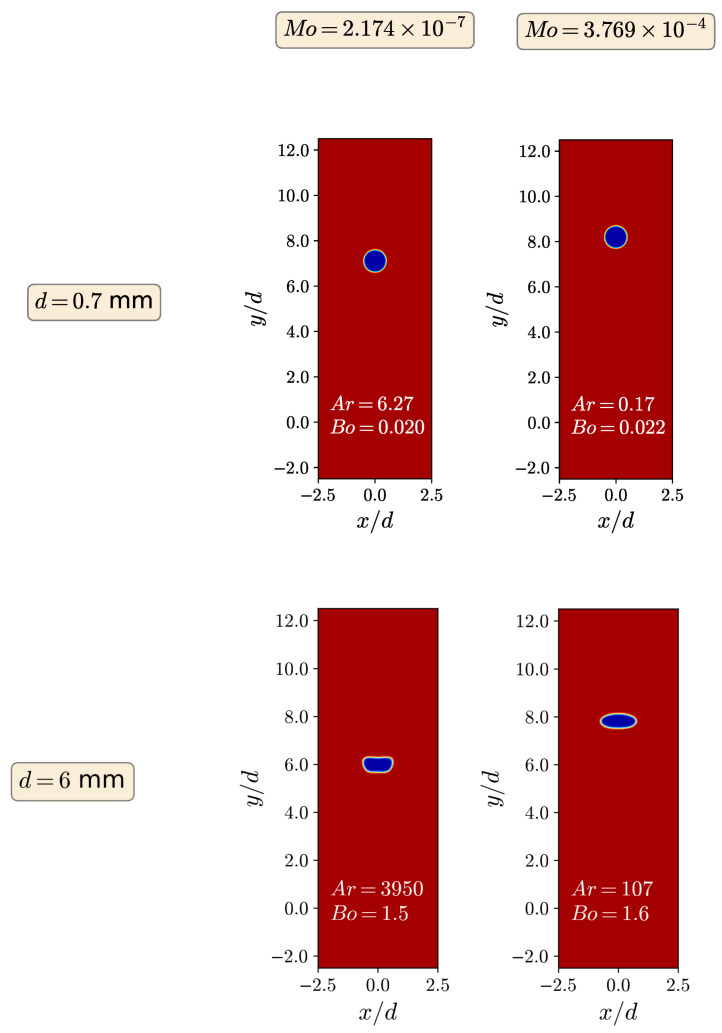
Contour plots of the fluid’s volume fraction for $Mo=2.174\times 10^{-7}$ (**left**) and $Mo=3.769\times 10^{-4}$ (**right**) with d=0.7mm (**top**) and d=6mm (**bottom**).

Figure 3 illustrates the contour plots of the steady-state bubble volume fraction within the Newtonian liquid for two distinct diameters, specifically, d=0.7 mm and d=6 mm, along with the corresponding two Mo numbers. As expected, the smaller bubbles, having low values of Ar and Bo, are perfectly spherical. As Ar and Bo dimensionless numbers rise, the effect of gravitational and inertia forces appears which affects the bubble shape. As the bubble diameter increases, Ar also enhances, and for Ar=107, the bubble changes from spherical to oblate-spheroid, as it can be seen in Figure 3 (the case with d=6 mm, $Mo=3.769\times 10^{-4}$). When Ar increases even more, the bubble shape turns to a more complex oblate, having flat front and back sides, with an indentation of the front side, as it is shown in Figure 3 (the case with d=6 mm, $Mo=2.174\times 10^{-7}$). The bubble shape in all the cases is in good agreement with the Newtonian cases displayed and discussed by Tsamopoulos et al. [7].

### 4.2. Buoyancy-Driven Rise of a Bubble through a Viscoelastic Shear-Thinning Fluid

**Figure 4:**
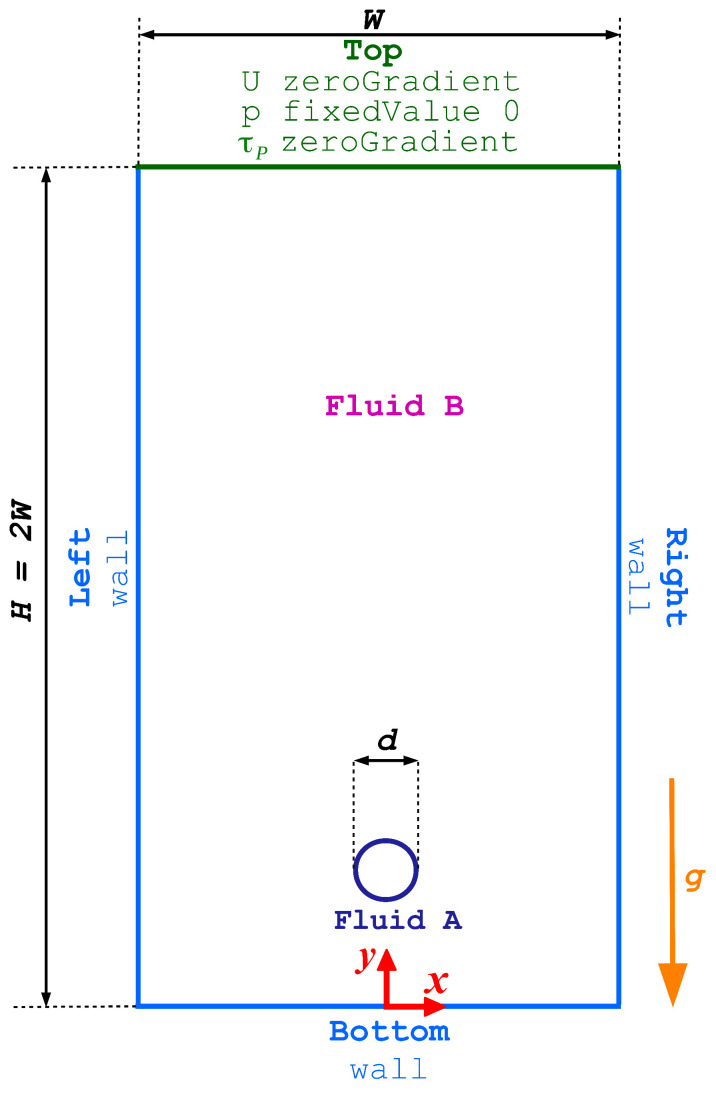
The geometry and boundary conditions for the buoyancy-driven rise of a bubble through a viscoelastic shear-thinning fluid.

In this section, we focus on the buoyancy-driven rise of a bubble through a viscoelastic shear-thinning fluid on a bounded domain. Figure 4 presents a schematic representation of the computational domain used for simulating the buoyancy-driven rise of a bubble through a viscoelastic shear-thinning fluid. In the simulations, the dimensions of the domain are fixed with a constant height *H* and width *W*. Specifically, *H* is equal to 2W and measures 16 cm. The mesh resolution for each simulation is chosen such that the initial diameter of the bubble is covered by 80 cells in both the *x* and *y* directions. In addition to the domain size, the boundary conditions employed in this case are distinct from those used in the buoyancy-driven rise of a bubble through a Newtonian fluid presented in Section 4.1. As depicted in Figure 4, the bottom, left, and right boundaries are treated as solid walls, where a *fixed value* velocity boundary condition of zero is applied, a *zeroGradient* pressure condition is enforced and a *linear extrapolation* of the polymeric stress components to the wall is carried out. On the other hand, the top boundary is subjected to a *zeroGradient* condition for both the velocity and polymeric stress components, and a *fixed value* pressure condition of zero. Furthermore, the fluid’s volume fraction α is subjected to a *zeroGradient* boundary condition at all the four boundaries.

Table 2 presents the fluid properties of fluids A and B used in the simulation of the buoyancy-driven rise of a bubble through a viscoelastic shear-thinning fluid. The viscoelastic fluid B is modeled using the Giesekus constitutive equation and fluid A is defined with dynamic viscosity and density properties similar to those of air. These properties closely resemble those utilized in the case study conducted by Ji et al. [9].

In this case study, two of the dimensionless groups which emerge from the governing equations are the Reynolds number, which is the ratio of the inertia forces to the viscous forces, $Re=\rho_{B}U^{*}d/\eta_{0}$; and the Weissenberg number, that is the magnitude of elastic forces with respect to the viscous forces, $Wi=\lambda U^{*}/R$, in which λ is the relaxation time, and the bubble terminal velocity over the bubble radius ($U^{*}/R$) is the characteristic shear rate [9].

**Figure 5:**
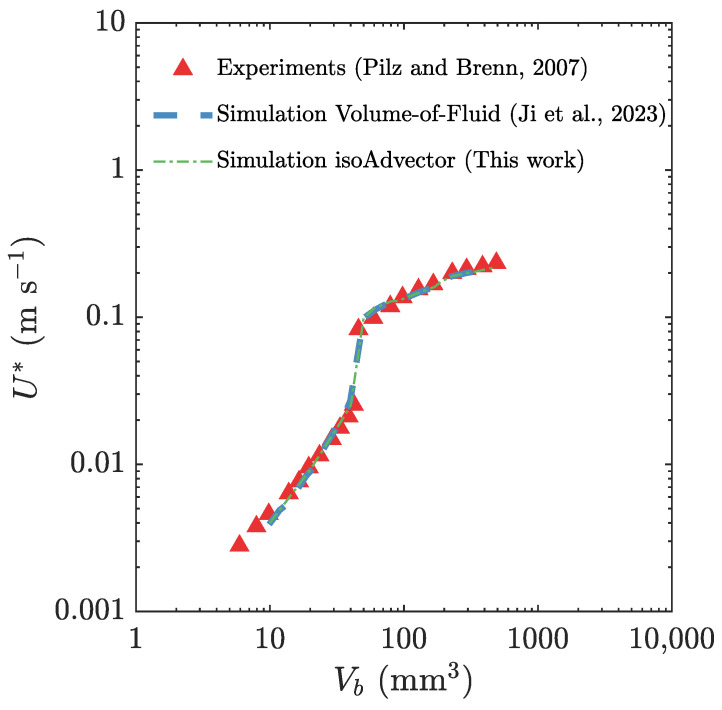
Comparison of our predicted bubble rise velocity with experimental data reported by Pilz and Brenn [74] and numerical simulation results by Ji et al. [9]. The comparison is conducted for various initial bubble volumes as they ascend through a shear-thinning viscoelastic fluid described by the Giesekus constitutive model.

Multiple simulations were conducted using the newly developed MVP-RIA algorithm, spanning a range of initial bubble volumes ($V_{b}$) from 10 mm${}^{3}$ to 400 mm${}^{3}$. This range encompasses both the subcritical bubble volume of 40 mm${}^{3}$ and the supercritical bubble volume of 50 mm${}^{3}$, with the velocity transition occurring in between. This specific volume range is referred to as the bubble critical volume. Figure 5 depicts the steady-state terminal velocity $U^{*}$ of the bubble as a function of the initial bubble volume $V_{b}$. The simulations in this study were conducted with the newly developed MVP-RIA algorithm, and the results obtained were compared with the simulations conducted by Ji et al. [9] using the VOF method, as well as with experimental data reported by Pilz and Brenn [74]. The results achieved using the newly developed algorithm exhibit excellent agreement with both the VOF method and the experimental data.

**Figure 6:**
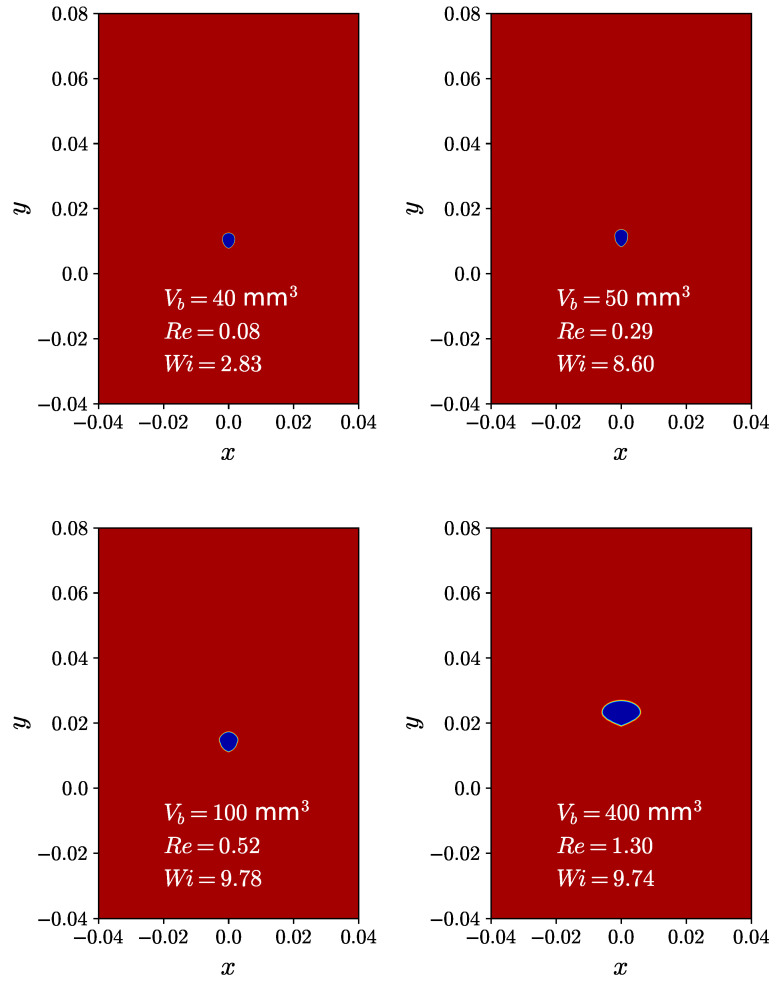
Contour plots of the fluid’s volume fraction for initial bubble volumes of $V_{b}=40,\;50,\;100$ and 400mm${}^{3}$ as they ascend through a shear-thinning viscoelastic fluid described by the Giesekus constitutive model.

In the case of Newtonian fluids, the dominant forces that influence the shape of a bubble are viscosity, surface tension, and inertia. These forces collectively lead to the formation of spherical, ellipsoidal, or spherical-cap bubble shapes [9,75,76]. The deformation of bubbles in viscoelastic fluids is primarily driven by the effects of viscoelasticity. Figure 6 illustrates the impact of the initial bubble volume on the resulting bubble shape in a steady state. Contour plots of the fluid volume fraction are presented for simulations with different initial bubble volumes, namely $V_{b}$ = 40, 50, 100, and 400 mm${}^{3}$. The bubble shape at the subcritical volume is prolate, while at the supercritical volume, it is an inverted teardrop with a cusp [9]. In viscoelastic fluids, when the normal stress is relatively small compared to the surface tension, the bubble shape remains spherical, similar to that observed in Newtonian fluids. As the normal stress becomes dominant over the surface tension, the bubble shape transitions to a prolate or teardrop shape, and a cusp may appear at the tail of the bubble [9].

**Figure 7:**
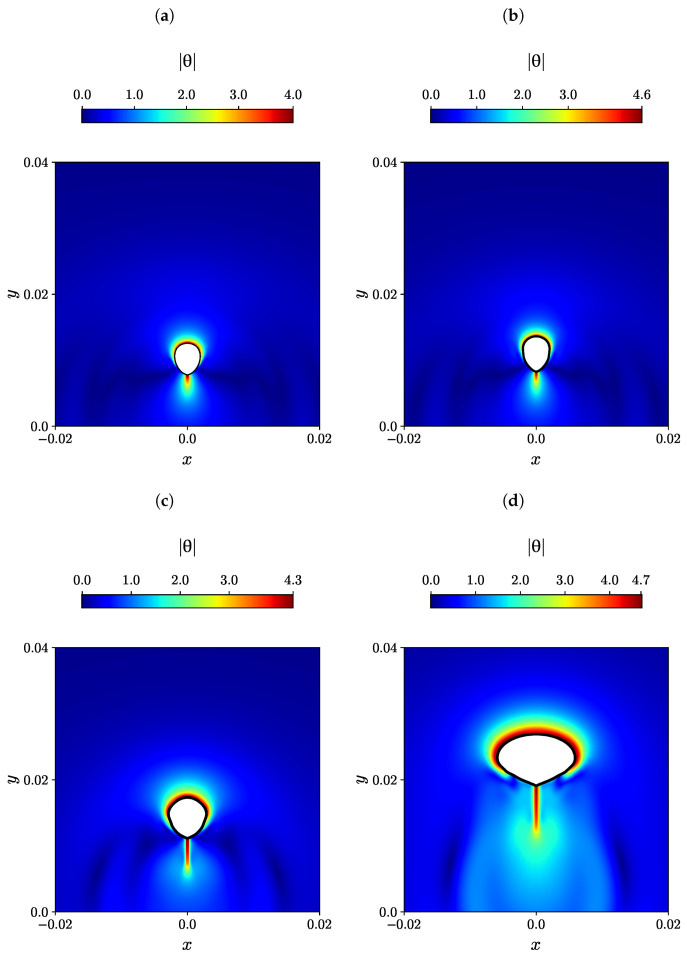
Contour plots illustrating the magnitude of the natural logarithm θ of the conformation tensor are presented for various initial bubble volumes, including (**a**) $V_{b}=40$, (**b**) $V_{b}=50$, (**c**) $V_{b}=100$, and (**d**) $V_{b}=400$ mm${}^{3}$, when they ascend through a shear-thinning viscoelastic fluid described by the Giesekus constitutive model.

In viscoelastic fluids, such as polymers, the presence of polymeric macromolecules results in entanglements between them. When the fluid flows, these macromolecules are extended in the direction of the flow, generating elastic stress. This elastic stress tends to relax when the fluid flow comes to cease. Therefore, the normal viscoelastic stress is closely connected to the conformation of polymeric macromolecules. The magnitude of the natural logarithm of the conformational tensor θ provides a measure of the extent of deformation of the polymeric macromolecules [9,77,78]. Figure 7 illustrates the contour plots of the natural logarithm θ of the conformation tensor for different bubble volumes, including the subcritical volume $V_{b}=40$, supercritical volume $V_{b}=50$, as well as higher volumes of $V_{b}=100$ and 400 mm${}^{3}$. The magnitude of θ is highest in the front side of the bubble and the wake region at the back, showing a uniform distribution in the front side, indicating compression and attachment of polymeric macromolecules in that region. Conversely, in the back of the bubble, the polymeric macromolecules are extended toward the tail end. These findings align with the simulations conducted by Ji et al. [9]. The value of θ in the subcritical bubble volume is lower compared to the supercritical bubble volume, indicating that the polymeric macromolecules experience greater stretching in the wake region of the supercritical bubble volume.

### 4.3. Buoyancy-Driven Rise of a Bubble through an Elastoviscoplastic Fluid

In this section, we investigate the buoyancy-driven rise of a bubble with volume $V_{b}$, which begins to move from a stationary position within an unbounded elastoviscoplastic medium and reaches a steady-state terminal velocity $U^{*}$. The dimensions of the computational domain and boundary conditions employed in this study are similar to the ones shown in Figure 1, with the additional conditions for the polymeric extra-stress tensor.

For the description of the elastoviscoplastic material, we used the same parameters described in Moschopoulos et al. [79], where the Saramito–Herschel–Bulkley constitutive model [52] is used to characterize the rheology of Fluid B. For all the simulations conducted in this study, an aqueous Carbopol solution with a concentration of 0.1% was employed. The dimensionless numbers that arise for this problem are the Archimedes number (Ar), measuring the ratio of gravity force to the viscous force, the Bond number (Bo), being the ratio of gravity force to capillarity force, the Bingham number (Bn), given by the ratio of yield stress to gravity force, and lastly, the elastogravity number (Eg), which is the ratio of gravity force over elasticity force. These non-dimensional numbers are defined as

(11) $$Ar=\frac{\rho gR_{eff}}{k{\left(\frac{\sqrt{gR_{eff}}}{R_{eff}}\right)}^{n}},Bo=\frac{\rho gR_{eff}^{2}}{\sigma },Bn=\frac{\tau_{0}}{\rho gR_{eff}},Eg=\frac{\rho gR_{eff}}{G},$$

where *G* is the elastic modulus of the material and $R_{eff}$ is the effective bubble radii defined as $R_{eff}={\left(3V_{b}/4\pi \right)}^{1/3}$. This study encompasses four scenarios characterized by different effective bubble radii, namely 0.004, 0.0083, 0.0107, and 0.0163 m, as outlined in Table 3.

**Figure 8:**
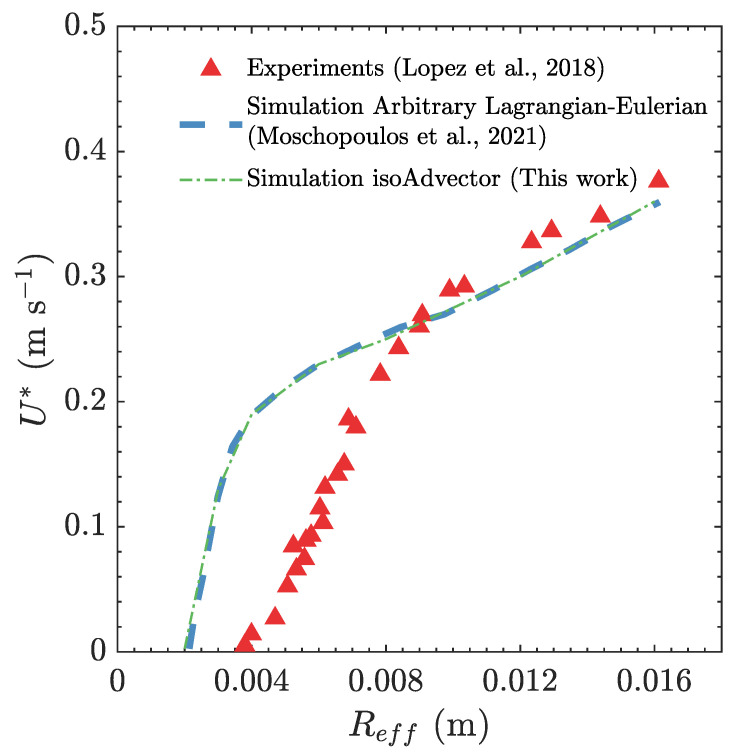
Bubble steady-state velocity $U^{*}$ as a function of the bubble initial radius $R_{eff}$. The dot-dashed line represent the results obtained with our MVP-RIA algorithm, the dashed line represent the results obtained with the ALE algorithm from Moschopoulos et al. [79] while the symbols represent the experimental data from Lopez et al. [80].

Figure 8 illustrates the steady-state terminal velocity $U^{*}$ of an air bubble rising through an elastoviscoplastic fluid for different effective bubble radius $R_{eff}$. The plot includes experimental data from Lopez et al. [80], results from the Arbitrary Lagrangian-Eulerian (ALE) simulations performed by Moschopoulos et al. [79], and our MVP-RIA calculations. The terminal velocity of the air bubble in both simulations exhibits a close agreement. For effective bubble radii $R_{eff}<0.008$ m, both simulations demonstrate higher bubble velocities with a concave shape function. In contrast, the experimental results show an almost linear bubble bulk velocity. For a detailed explanation of the larger deviations between the experimental and predicted terminal velocity of smaller bubbles the reader is referred to Moschopoulos et al. [79], see Section 5.3 therein. The steady-state bubble velocity of the simulations and the experiment intersect at $R_{eff}\approx 0.008$ m, and thereafter the experiment and the simulations are in good agreement, both have a linear trend with respect to $R_{eff}$.

**Figure 9:**
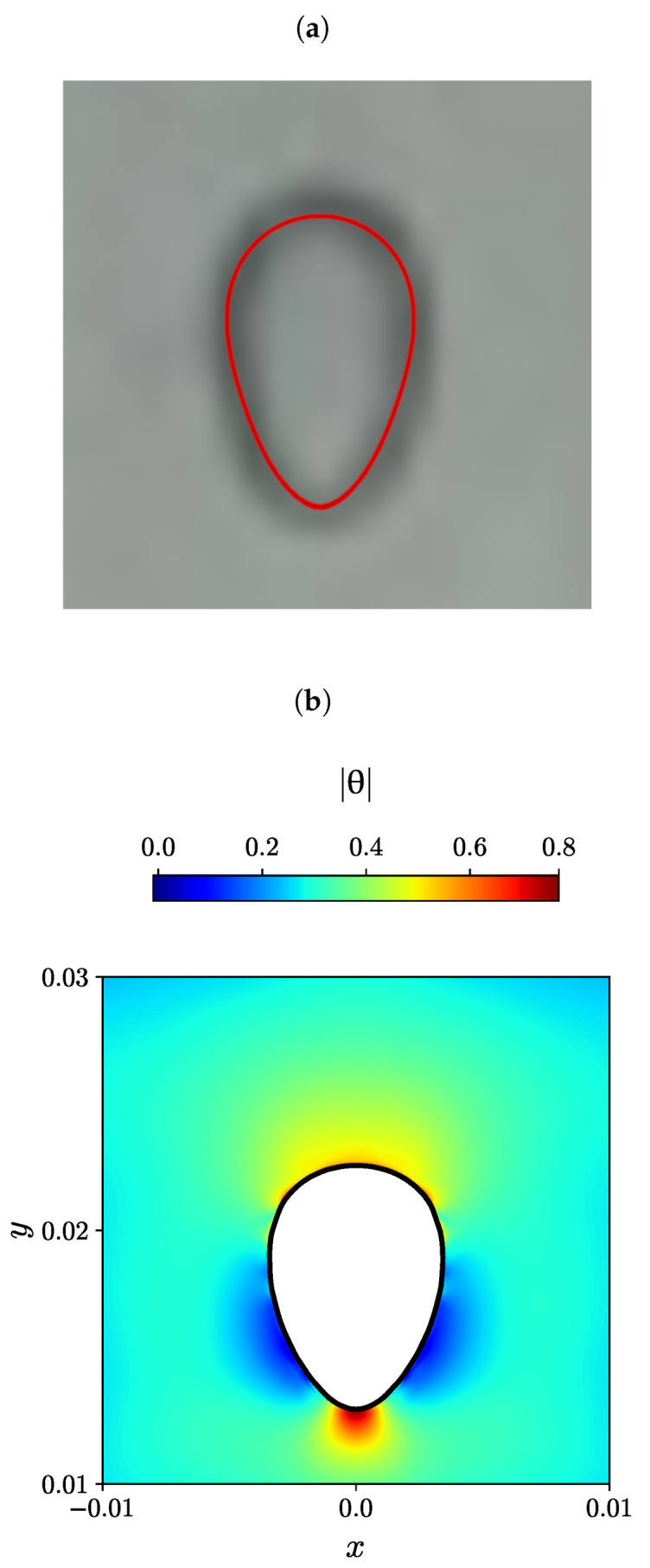
(**a**) Experimental bubble shape by Lopez et al. [80] (black and white), and the numerical simulation of Moschopoulos et al. [79] (red line), (**b**) Contour plots of the natural logarithm θ of the conformation tensor for $R_{eff}=0.004\;$m obtained with the newly developed MVP-RIA algorithm.

**Figure 10:**
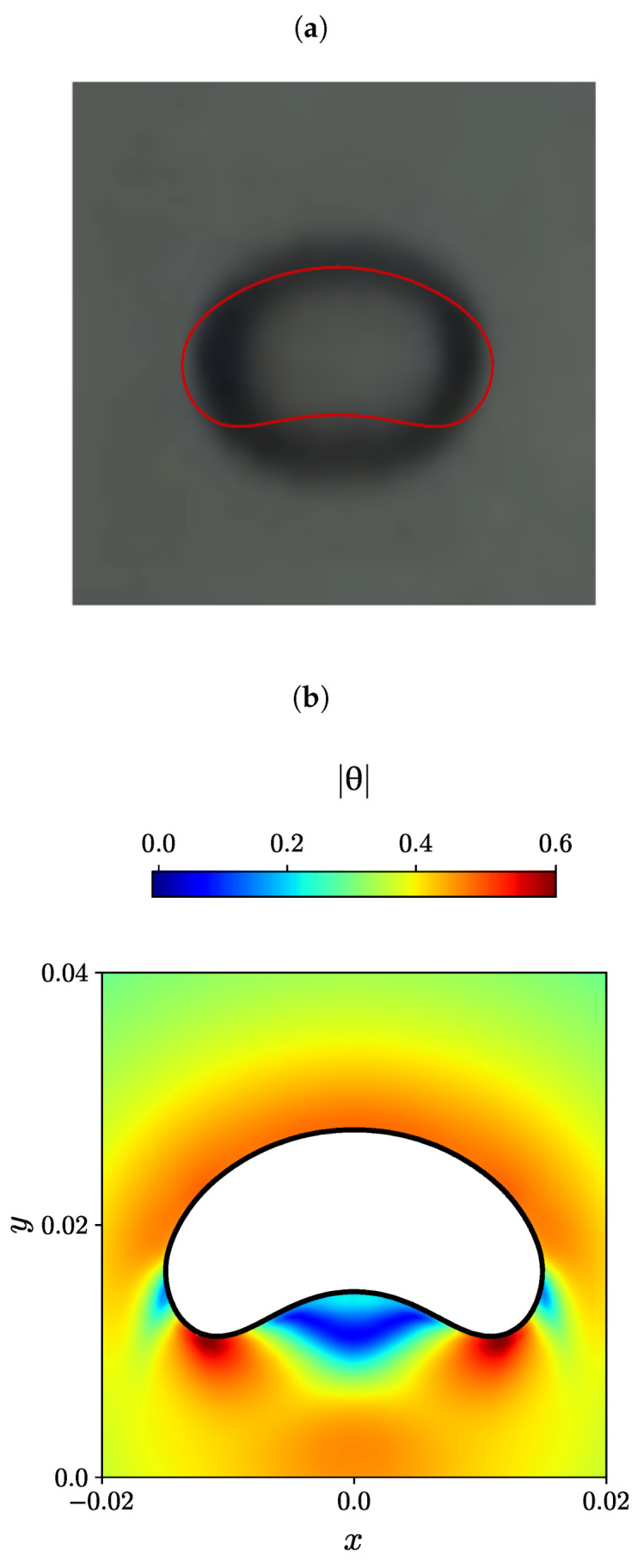
(**a**) Experimental bubble shape by Lopez et al. [80] (black and white), and the numerical simulation of Moschopoulos et al. [79] (red line), (**b**) Contour plots of the natural logarithm θ of the conformation tensor for $R_{eff}=0.0107\;$m obtained with the newly developed MVP-RIA algorithm.

**Figure 11:**
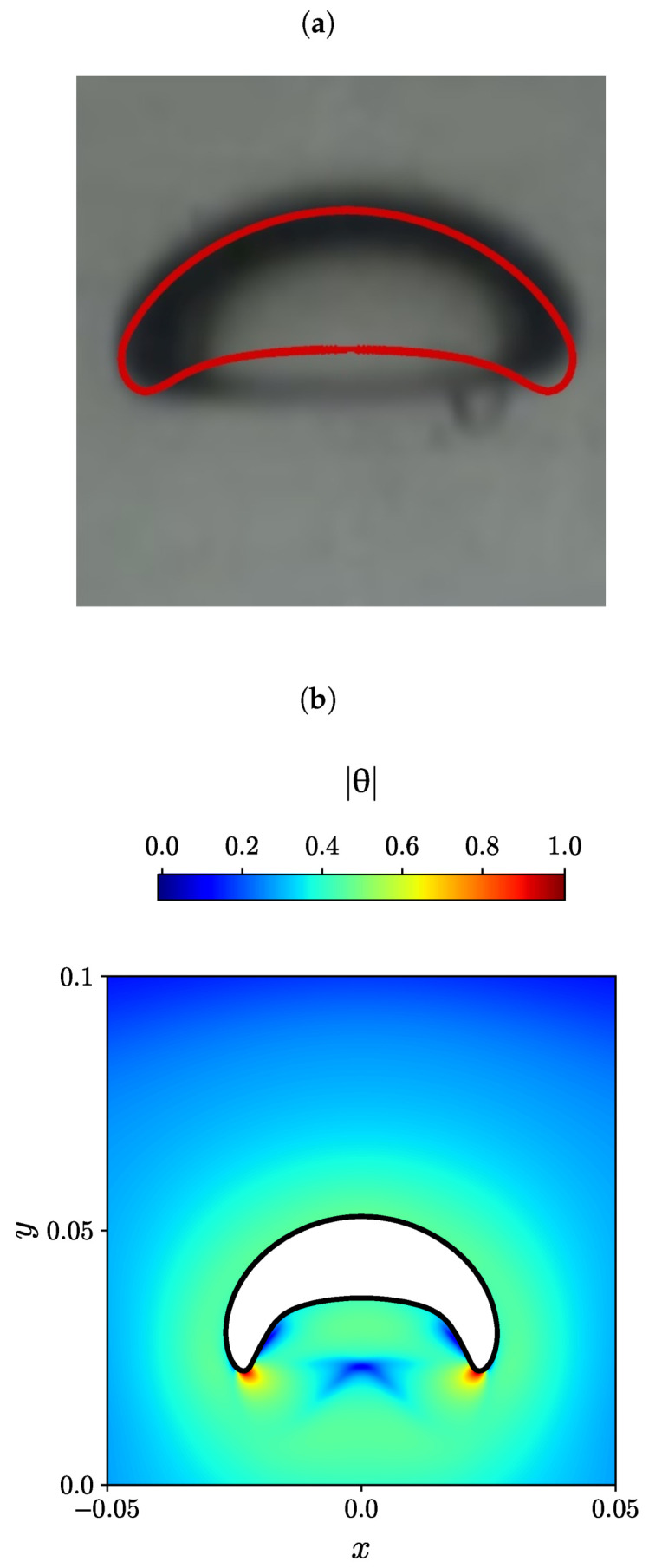
(**a**) Experimental bubble shape by Lopez et al. [80] (black and white), and the numerical simulation of Moschopoulos et al. [79] (red line), (**b**) Contour plots of the natural logarithm θ of the conformation tensor for $R_{eff}=0.0163\;$m obtained with the newly developed MVP-RIA algorithm.

The experimental steady-state bubble shape is depicted in black and white in Figure 9a, Figure 10a and Figure 11a, as reported by Lopez et al. [80] for three different effective bubble radii, specifically, $R_{eff}=$ 0.004, 0.0107, and 0.0163 m. In addition, the numerically predicted bubble shape by Moschopoulos et al. [79] is superimposed with a red line. On the bottom of each figure (panels (b)), the numerically predicted air bubble shape obtained using the MVP-RIA algorithm developed in this study is represented by a solid black line. The contour plots of the natural logarithm θ of the conformation tensor are also displayed.

The steady-state bubble shape for the case with $R_{eff}=0.004$ m exhibits a similar appearance in the experiment conducted by Lopez et al. [80], the ALE simulation by Moschopoulos et al. [79] (Figure 9a), and our MVP-RIA simulation (Figure 9b). For this particular case, the air bubble in the elastoviscoplastic fluid exhibits an elongated shape along the axial coordinate, resembling a reverse teardrop shape with a gentle tip. The dominance of elasticity and capillary forces in very small air bubbles within the elastoviscoplastic fluid restricts the extent of bubble deformation. The contour plot of the magnitude of the natural logarithm θ in Figure 9b indicates that in the front side of the bubble, there is also a uniform distribution of this quantity with a value larger than the mean. The maximum value of θ appears at the bubble tail end. The minimum values of θ are in a relatively large area at the bubble sides, closer to its end tail.

For the case of the bubble effective radius equal to $R_{eff}=0.0107$ m, the bubble shape forms a spherical-cap shape. In this case, the bubble is stretched laterally, and two tails emerge. The contour plot of the magnitude of the natural logarithmic θ in Figure 10b shows that the maximum value occurs at the two tails of the bubble, and the minimum value of |θ| is being transferred from the bubble sides to the back side of the bubble in the area between the two tails.

In the case of the highest effective radius $R_{eff}=0.0107\;$m, we can observe a more laterally stretched bubble with two sharper tails, as shown in Figure 11. The trend is similar in the experiment [80], the ALE simulation [79], and with our MVP-RIA algorithm, although the latter shows a more arc-shaped bubble, with sharper tails. Here, we observed a more uniform area with the same value of |θ| around the bubble, while the region showing the maximum value of |θ| has shrunk into small spots near the two sharp tails, and the minimum |θ| region is in the inner lateral sides of the two tails.

Lastly, the disparities observed in the bubble contour shape obtained through the numerical models, when contrasted with the experimental counterparts in Figure 10 and Figure 11, have been attributed by Moschopoulos et al. [79] (as discussed in Section 5.2.1 of their work) to the absence of rheological data pertaining to elongational flow. This absence leads to an underestimation of the material’s elastic behavior.

## 5. Conclusions

In conclusion, the motion of air bubbles within a liquid has been shown to have significant implications in various aspects, specifically, bubbles affect the viscosity, flow behavior, and overall rheological properties of non-Newtonian fluids, particularly elastoviscoplastic fluids. This study introduces a novel interface-capturing method specifically designed for multiphase viscoelastic fluid flow simulations. The developed algorithm combines the geometric volume of fluid (isoAdvector) approach with a reconstructed distance function (RDF) to accurately determine interface curvature. To further enhance accuracy and robustness, a piecewise linear interface construction (PLIC) scheme is incorporated in conjunction with the RDF-based interface reconstruction. The proposed method offers improved capabilities for capturing interfaces in complex viscoelastic fluid flow scenarios, providing a valuable tool for studying and analyzing multiphase systems involving non-Newtonian fluids.

The multiphase viscoelastic PLIC-RDF isoAdvector (MVP-RIA) algorithm has been successfully validated through simulations of the buoyancy-driven rise of a bubble in fluids with diverse rheological characteristics. The algorithm was initially employed to investigate the behavior of a bubble rising in a Newtonian fluid within an unbounded domain. The results obtained from these simulations exhibit remarkable agreement with experimental data, providing accurate predictions of the bubble shape and velocity. Subsequently, by incorporating the effects of viscoelasticity, shear-thinning behavior, and elastoviscoplasticity, the MVP-RIA algorithm opens up new possibilities for exploring the influence of rheological complexity on bubble dynamics. The MVP-RIA algorithm was further extended to investigate the buoyancy-driven rise of a bubble in a viscoelastic shear-thinning fluid utilizing the Giesekus constitutive model. The simulations revealed a remarkable change in the bubble shape as the influence of normal stress became dominant over surface tension. Specifically, the bubble shape transitioned from a spherical, ellipsoidal, or spherical-cap shape observed in the Newtonian fluid case to a prolate or teardrop shape, often characterized by a cusp at the bubble tail. This observation highlights the significant impact of viscoelasticity on the deformation behavior of bubbles in non-Newtonian fluids. The presence of viscoelasticity introduces additional forces and rheological complexities, leading to distinct bubble shapes and dynamics compared to Newtonian fluids. The ability of the MVP-RIA algorithm to accurately capture these complex phenomena demonstrates its effectiveness in simulating the behavior of bubbles in viscoelastic shear-thinning fluids. In the final phase of our study, the MVP-RIA algorithm was utilized to investigate the buoyancy-driven rise of a bubble in an unbounded elastoviscoplastic medium, employing the Saramito–Herschel–Bulkley constitutive equation. For very small air bubbles immersed in the elastoviscoplastic fluid, the dominance of elasticity and capillary forces imposes limitations on the extent of bubble deformation. Consequently, the deformation is primarily confined to the axial direction, resulting in a stretched, reverse teardrop shape with a mild tip along its trajectory. However, as the bubble volume increases, lateral stretching becomes more pronounced, leading to the emergence of two tails. This evolution ultimately gives rise to a highly elongated bubble shape with sharper tails.

The successful validation of the MVP-RIA algorithm paves the way for future investigations into a wide range of practical applications, including industrial processes, materials engineering, and biomedical research.

## Figures and Tables

**Table 1:** Fluid properties for simulation of the buoyancy-driven rise of a bubble in a Newtonian fluid.

Fluid	Dynamic Viscosity	Density	Surface Tension	Morton Number
	$\eta_{S}$ (Ns/m${}^{2}$)	ρ (kg/m${}^{3}$)	σ (N/m)	Mo $=g\eta_{S}^{4}/\rho \sigma^{3}$
B-1	$9.45\times 10^{-3}$	1153.8	0.06782	$2.174\times 10^{-7}$
B-2	$6.01\times 10^{-2}$	1208.5	0.06550	$3.769\times 10^{-4}$
A	$1.48\times 10^{-5}$	1		

**Table 2:** Fluid properties for simulation of the buoyancy-driven rise of a bubble in a viscoelastic shear-thinning fluid.

Fluid	Solvent Viscosity	Polymer Viscosity	Density	Surface Tension	Relaxation Time	Mobility Factor
	$\eta_{s}$ (Ns/m${}^{2}$)	$\eta_{p}$ (Ns/m${}^{2}$)	ρ (kg/m${}^{3}$)	σ (N/m)	λ (s)	α
B	$1.0\times 10^{-3}$	1.511	1000.90	0.076	0.207	0.6
A	$1.7\times 10^{-5}$		1.25			

**Table 3:** Dimensionless numbers employed for the simulation of the buoyancy-driven rise of a bubble through an elastoviscoplastic fluid.

$R_{\mathrm{eff}}$ [m]	Ar	Bn	Bo	Eg
0.004	3.610	0.119	2.150	0.971
0.0083	8.929	0.057	9.347	2.024
0.0107	12.090	0.044	15.385	2.597
0.0163	20.410	0.029	35.704	3.956

## Data Availability

The data presented in this study are available on request from the corresponding author. The data are not publicly available due to privacy.

## Abbreviations

The following abbreviations are used in this manuscript:

ADI	Alternating-Direction Implicit
ALE	Arbitrary Lagrangian Eulerian
BiCGStab	Bi-Conjugate Gradient-Stable Algorithm
CSF	Continuum Surface Force
CUBISTA	Convergent and Universally Bounded Interpolation Scheme
	for the Treatment of Advection
FTM	Front-Tracking Method
GAMG	Geometric-Algebraic Multi-Grid
IBM	Immersed Boundary Method
IFEM	Immersed-Finite-Element Method
ILU	Incomplete Lower-Upper
LSM	Level-Set Method
MAC	Marker-And-Cell
MULES	Multidimensional Universal Limiter with Explicit Solution
MVP-RIA	Multiphase Viscoelastic PLIC-RDF isoAdvector
OpenFOAM	Open Source Field Operation and Manipulation
PFM	Phase Field Method
PIMPLE	Mixture of PISO and SIMPLE
PISO	Pressure Implicit with Splitting of Operator
PLIC	Piecewise Linear Interface Construction
RDF	Reconstructed Distance Function
SIMPLE	Semi-Implicit Method for Pressure Linked Equations
VOF	Volume-Of-Fluid
**Nomenclature**	
**Physical and mathematical quantities**	
**u**	Velocity vector
ρ	Density
*p*	Pressure
**g**	Gravity acceleration
$f_{s}$	Surface tension force
τ	Stress tensor
$\tau_{S}$	Newtonian (Solvent) stress tensor
$\tau_{P}$	Polymeric extra-stress tensor
$\eta_{S}$	Solvent dynamic viscosity
$\eta_{P}$	Polymeric dynamic viscosity
α	Mobility parameter
λ	Relaxation factor, relaxation time
ε	Extensibility parameter
$\sigma_{D}$	Deviatoric part of stress tensor
$\bar{\sigma }$	Second invariant of the deviatoric stress tensor
I	Identity tensor
$\tau_{0}$	Yield stress
${\overset{\;□}{\tau }}_{P}$	Gordon-Schowalter derivative
ζ	Non-affine deformation parameter
${\overset{▿}{\tau }}_{P}$	Upper-convective time derivative of the polymeric extra-stress tensor
A	Conformation tensor
γ	Volume fraction
**u** ${}_{r}$	Relative velocity vector of two fluids
σ	Surface tension coefficient
$U^{*}$	Calculated rise velocity
θ	Natural logarithm of the conformation tensor
*k*	Consistency index
*n*	Shear-thinning exponent
*G*	Elastic modulus of the material
**Geometrical parameters**	
*W*	Domain width
*H*	Domain height
*d*	Initial bubble diameter
*R*	Initial bubble radius
$V_{b}$	Initial bubble volume
$R_{eff}$	Effective bubble radii
**Non-dimensional numbers**	
Ar	Archimedes number
Bo	Bond (Eötvös) number
Mo	Morton number
Re	Reynolds number
Wi	Weissenberg number
Bn	Bingham number
Eg	Elastogravity number
**Operators**	
∇	Gradient
∇.	Divergence
$\frac{D(.)}{Dt}$	Total time derivative
tr(.)	Trace operator
max(.,.)	Maximum operator
${\left(.\right)}^{T}$	Transpose operator
:	Double dot product
